# Supplementary material for: RNA-Seq Insight into the Impact and Mechanisms of Methyl Donor and Glycine Betaine Osmoprotectant on Polyketide Secondary Metabolism in Monascus purpureus M1
Source: J Fungi (Basel). 2025 Apr 1;11(4):273. doi: 10.3390/jof11040273 (PMC12028682; doi:10.3390/jof11040273)
Supplement: Supplementary file 1 [file jof-11-00273-s001.zip › jof-3521396-supplementary.pdf]

Table S1. Primer sequences for the key genes in M. *purpureus* M1.

Gene	Primer sequences(5′to3′)	Length (bp)	Tm Value	PCR Product Length (bp)	Description
mokA-F	GACCTCGGTCATCTTGGC	18	57	78	polyketide synthase
mokA-R	TTGTTCCAAGCGGTCTTC	18	54	78	
mokB-F	AAACATCGTCACCAGTCT	18	53	78	polyketide synthase
mokB-R	CTAAGTCGGGCATCTACC	18	53	78	
mokC-F	CAAGCTGCGAAATACACCAAGCCTC	25	62	80	P450 monooxygenase
mokC-R	AGCCGTGTGCCATTCCTTGTTGTCC	25	60	80	
mokD-F	TTCATCTGCTGCTGGTAT	18	53	92	oxidoreductase
mokD-R	AACTTCTCACCGTCAATG	18	52	92	
mokE-F	ATCGCAGGTCACGCACATCCAAGTC	25	65	221	dehydrogenase
mokE-R	GTAAAGGCAGCCCGAGCAGCTTCAT	25	65	221	
mokF-F	GAGATCATAGTGGCCGACTGAA	22	60	190	transesterase
mokF-R	ACCGTCTCATCCAACCTCACGA	22	61	190	
mokG-F	CCAGGTAACCAACGGATTA	19	50	82	HMG-CoA reductase
mokG-R	GATCAGAGCAGTCACCAG	18	54	82	
mokH-F	CAGGAAATCTGGACTTACCCCATTG	25	58	123	transcription factor
mokH-R	TGTTGGATTGTTGTTGGAGATATAC	25	55	123	
mokI-F	ATGTTGAATGGCAATGATGG	20	60	135	efflux pump
mokI-R	CAGCGTGGGTGATGTATC	18	57	135	
mppA-F	TCCCGTTTCTTGGACGTGAG	20	59	132	C-11-ketoreductase
mppA-R	ACGTGCCATGGTTCTGTCTT	20	59	132	
mppB-F	CGTCTCGCCCGATAACTTCA	20	60	108	acyltransferase
mppB-R	TTGACAGACGGGTCGAAGTC	20	58	108	

mppC-F	CAGTCCTCGTCCCTTCCAGT	20	60	137	NADPH-dependent oxidoreductase
mppC-R	CCACGGTGAAGGATGTCGAG	20	58	137	
mppD-F	TCAACACGGGAGATGCTGTC	20	62	140	serine hydrolase
mppD-R	GCCAAAGGACAGGAGCAGAT	20	63	140	
mppE-F	CTTCCCGATGCCGTTGTGAT	20	60	99	enoyl reductase
mppE-R	CGTCTCGTGGATCATCTCGT	20	60	99	
mppG F	TCAACACGGGAGATGCTGTC	20	56	140	FAD-dependent
mppG R	GCCAAAGGACAGGAGCAGAT	20	59	140	oxidoreductase
mpp7 F	ATCGTCGGATCAGCGTCAC	19	59	148	acetylatransferase
mpp7 R	CGGCTGTTATAGGGTGGC	18	57	148	
mppR1-F	TCTGCAGTATGCCATGTGGG	20	59	123	transcription factor
mppR1-R	ATGGCACCGTCACTTAGCTC	20	55	123	
mppR2-F	ACGAAACCCTCCATGACACC	20	59	138	transcription factor
mppR2-R	TGCAGACAGCCTTGTGGTAG	20	59	138	
MpPKS5-F	TGTCCGACGAGTTTCTGCAA	20	58	134	NR-PKS
MpPKS5-R	TATCAACGCTGCTTGGGCAT	20	60	134	
MpFasA2-F	ATGGATCGCCCGATCTTGTC	20	59	129	FAS subunit alpha
MpFasA2-R	CTTTGTCGAGTCCGCTGGAT	20	59	129	
MpFasB2-F	CCTCCAGGGATTACAACCCG	20	58	131	FAS subunit beta
MpFasB2-R	ATTCAATGCCAGGTGCTCCA	20	58	131	
GADPH-F	CCGTATTGTCTTCCGTAAC	19	55	114	Reference gene
GADPH-R	GTGGGTGCTGTCATACTTG	19	56	114	
laeA F	ACTCGTAGCGGATGTAAGA	19	55	105	global regulator
laeA R	CCGTGCTTGGTAGATGTG	18	55	105	
brlA F	ATGTCAGGGTGGCGTGAAGT	20	60	187	asexual development

brIA R	CCTGAACTGTACCTGCTTGAT	21	56	187	
wetA F	ATGTGTTATATTCCCCGGGA	20	60	174	asexual development
wetA R	TTAGCAGAGTGCGGCCTCGAG	21	62	174	

Figure S1. Protein-protein interaction network on day 2 of GB treatment

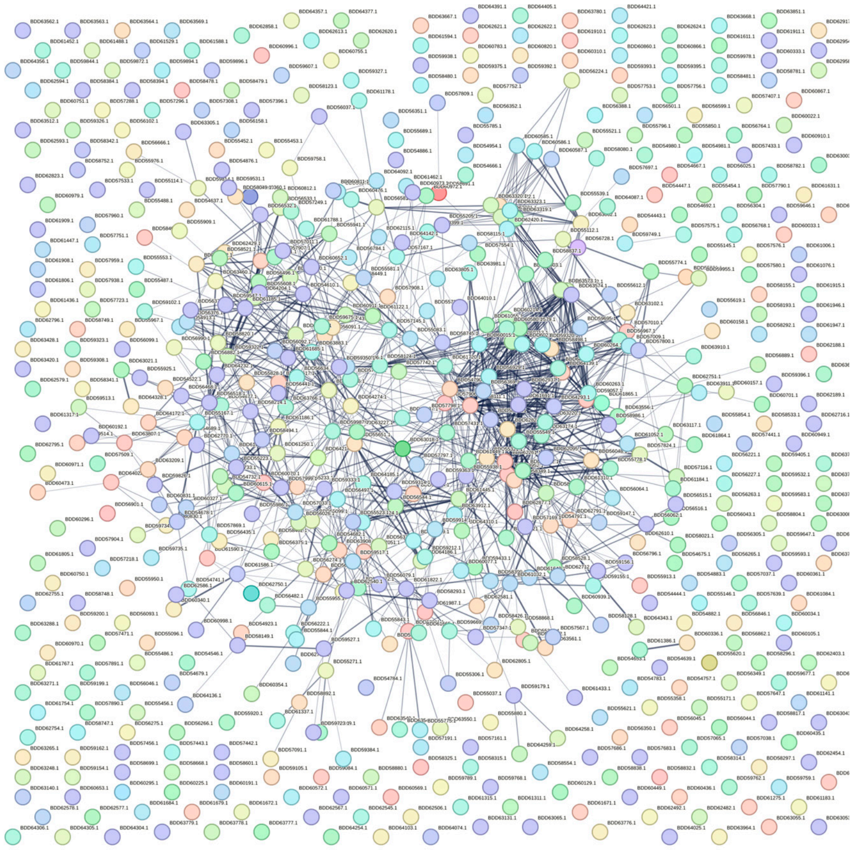

Figure S2. Protein-protein interaction network on day 12 of GB treatment

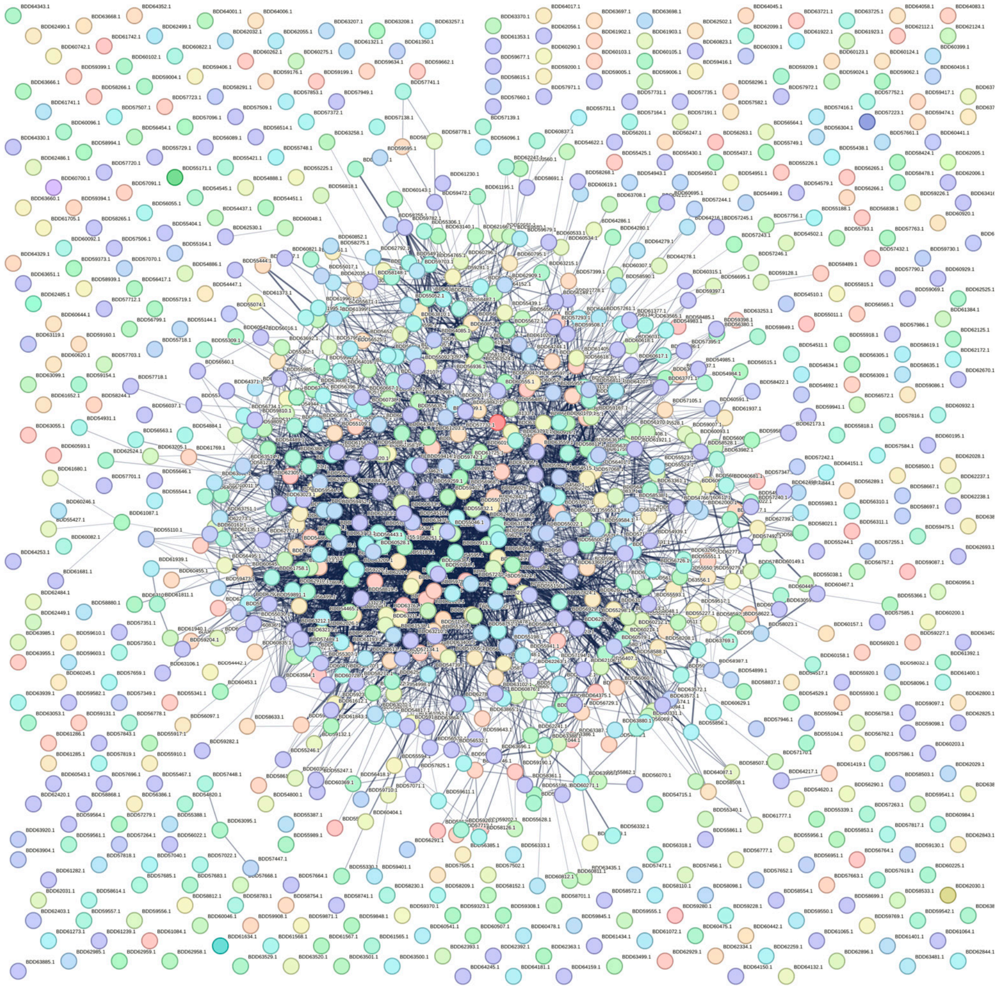

Table S2. The top 10 hub genes identified in the GB-treated group on Day 2, based on differential expression proteins, were determined using the CytoHubba plugin.

Category	Degree	MNC	MCC	EPC	Closeness	Betweenness	Bottleneck	Radiality
HUB gene TOP 10	**BDD58111.1**	**BDD58111.1**	**BDD58111.1**	**BDD58111.1**	**BDD58111.1**	**BDD58111.1**	BDD63018.1	**BDD58111.1**
	**BDD54790.1**	**BDD54790.1**	**BDD56929.1**	**BDD54790.1**	**BDD54790.1**	**BDD54790.1**	**BDD58111.1**	**BDD54790.1**
	**BDD56929.1**	**BDD56929.1**	BDD60264.1	**BDD56929.1**	**BDD61691.1**	BDD63018.1	**BDD54790.1**	BDD63018.1
	**BDD61691.1**	**BDD61691.1**	BDD64293.1	**BDD61691.1**	**BDD56929.1**	BDD61449.1	BDD61720.1	**BDD61691.1**
	BDD62824.1	BDD62824.1	**BDD54790.1**	BDD64138.1	BDD63018.1	**BDD56929.1**	BDD61449.1	**BDD56929.1**
	BDD58374.1	BDD64293.1	BDD60015.1	BDD62824.1	BDD62824.1	**BDD61691.1**	BDD55082.1	BDD61449.1
	BDD55082.1	BDD58374.1	BDD64139.1	BDD58374.1	BDD61449.1	BDD55082.1	BDD55524.1	BDD55549.1
	BDD64293.1	BDD64139.1	BDD64138.1	BDD60264.1	BDD58374.1	BDD56374.1	BDD63912.1	BDD62824.1
	BDD64139.1	BDD64138.1	BDD63574.1	BDD63574.1	BDD55549.1	BDD61720.1	**BDD61691.1**	BDD58374.1
	BDD64138.1	BDD60264.1	BDD63573.1	BDD64293.1	BDD55082.1	BDD56400.1	BDD56400.1	BDD63220.1

Table S3. The top 10 hub genes identified in the GB-treated group on Day 12, based on differential expression proteins, were determined using the CytoHubba plugin.

Category	Degree	MNC	MCC	EPC	Closeness	Betweenness	Bottleneck	Radiality
HUB gene TOP 10	**BDD58146.1**	**BDD58146.1**	**BDD57270.1**	**BDD61725.1**	**BDD58146.1**	**BDD58727.1**	**BDD58146.1**	**BDD58146.1**
	**BDD57270.1**	**BDD57270.1**	**BDD58189.1**	**BDD58146.1**	**BDD58727.1**	**BDD58146.1**	**BDD58727.1**	**BDD58727.1**
	**BDD58727.1**	BDD59620.1	BDD58251.1	**BDD57270.1**	BDD59620.1	BDD58133.1	BDD55046.1	BDD54560.1
	BDD59620.1	**BDD58727.1**	**BDD58146.1**	**BDD58727.1**	**BDD57270.1**	BDD59620.1	BDD58133.1	**BDD58189.1**
	BDD58811.1	**BDD58189.1**	BDD55046.1	BDD54515.1	**BDD61725.1**	BDD56478.1	BDD61691.1	BDD56026.1
	**BDD61725.1**	**BDD61725.1**	BDD54516.1	**BDD63559.1**	**BDD58189.1**	BDD61691.1	BDD57987.1	BDD59620.1
	**BDD58189.1**	BDD54515.1	BDD56436.1	**BDD58189.1**	BDD54515.1	BDD59621.1	BDD56478.1	**BDD61725.1**
	**BDD63559.1**	**BDD63559.1**	BDD55618.1	BDD56436.1	BDD54560.1	BDD56361.1	**BDD63559.1**	BDD54515.1
	BDD54515.1	BDD60916.1	BDD62020.1	BDD54595.1	**BDD63559.1**	**BDD57270.1**	BDD58208.1	**BDD63559.1**
	BDD54560.1	BDD54595.1	BDD58553.1	BDD58811.1	BDD56026.1	**BDD61725.1**	BDD59621.1	BDD58133.1

Genes in bold red represent those ranked in the top ten across eight algorithms; genes in bold blue represent those ranked in the top ten across

seven algorithms; and genes in bold green represent those ranked in the top ten across six algorithms.
Degree:node connect degree;MNC:Maximum Neighborhood Component;MCC:Maximal Clique Centrality;EPC:Edge Percolated Component;Betweenness:betweenness centrality

Table S4. Basic information of four heat shock proteins.

geneID	length	padj	Regulation	GeneSymbol	GO_BP	GO_CC	GO_MF	KO	Pathway
gene-MAP00_004934	2733	1.56E-154	Ups	HSP88	-	-	GO:0005524:ATP binding;GO:0016887:ATP hydrolysis activity;	K09489	-
gene-MAP00_004401	2648	1.08E-128	Ups	HSP90	GO:0006457: protein folding;	-	GO:0005524:ATP binding;GO:0016887:ATP hydrolysis activity;GO:0051082:unfolded protein binding;	K04079	ko04141 Protein processing in endoplasmic reticulum;;
gene-MAP00_003300	2387	4.06E-78	Ups	HSP70	-	-	GO:0005524:ATP binding;GO:0016887:ATP hydrolysis activity;	K03283	ko03040 Spliceosome;ko04141 Protein processing in endoplasmic reticulum;ko04144 Endocytosis;ko04213 Longevity regulating pathway - multiple species;;
gene-MAP00_007250	309	1.75E-08	Ups	HSP10	GO:0006457: protein folding;	-	GO:0005524:ATP binding;GO:0016887:ATP hydrolysis activity;	K04078	-
